# Corneal Stem Cells as a Source of Regenerative Cell-Based Therapy

**DOI:** 10.1155/2020/8813447

**Published:** 2020-07-20

**Authors:** Jasmin S. Nurković, Radiša Vojinović, Zana Dolićanin

**Affiliations:** ^1^Center for Regeneration and Rehabilitation, Novi Pazar, Serbia; ^2^Faculty of Medical Sciences, University of Kragujevac, Kragujevac, Serbia; ^3^Clinical Center Kragujevac, Kragujevac, Serbia; ^4^Department of Biomedical Sciences, State University of Novi Pazar, Novi Pazar, Serbia

## Abstract

In the past few years, intensive research has focused on corneal stem cells as an unlimited source for cell-based therapy in regenerative ophthalmology. Today, it is known that the cornea has at least two types of stem cells: limbal epithelial stem cells (LESCs) and corneal stromal stem cells (CSSCs). LESCs are used for regeneration of corneal surface, while CSSCs are used for regeneration of corneal stroma. Until now, various approaches and methods for isolation of LESCs and CSSCs and their successful transplantation have been described and tested in several preclinical studies and clinical trials. This review describes in detail phenotypic characteristics of LESCs and CSSCs and discusses their therapeutic potential in corneal regeneration. Since efficient and safe corneal stem cell-based therapy is still a challenging issue that requires continuous cooperation between researchers, clinicians, and patients, this review addresses the important limitations and suggests possible strategies for improvement of corneal stem cell-based therapy.

## 1. Introduction

The cornea represents the part of transparent tissue at the front of the eye. It poses a protective physical and biological barrier against the outside environment and gives a refractive power to concentrate light onto the retina. The thickest layer of the cornea, the corneal stroma, embodies a unique avascular connective tissue which constitutes approximately 90% of the cornea volume. Its highly organized extracellular matrix consists of tightly packed parallel collagen type I of V fibrils [1, 2]. The corneal stroma is maintained by the keratinocytes, which originate from the neural crest. In contrast to normal corneal development where the newly formed collagen fibers are quickly formed into a well-organized structure, corneal injury results in the formation of a disorganized opaque matric known as a corneal scar tissue [3] that reduces corneal transparency and may cause blindness [4, 5].

Considering that scarring involving the center of the cornea will cause significant visual loss and is mainly irreversible, the most common method of therapy is corneal transplantation from cadaveric donor. This method became widely accepted and successful because of tissue accessibility and immune privilege of the cornea. Despite this, the need for new corneal tissues has increased over the last few years since corneal grafts have had a failure rate of around 38%, mainly because of graft rejection [6, 7]. Thus, it is imperative to find new approaches for endothelial regeneration or replacement that may lead to better outcomes. The remarkable progress, which could sidestep the constraints of current treatments, has been made with the development of an autologous transplant of cultured endothelial cells into a patient's anterior chamber that can redesign the corneal tissue and with the generation of corneal stroma-like tissue developed from autologous stem cells [8].

With respect to the latter, in the past few years, intensive research has focused on corneal stem cells as a source of regenerative cell-based therapy. Today, it is known that the cornea has at least two types of stem cells: limbal epithelial stem cells (LESCs) and corneal stromal stem cells (CSSCs). LESCs are used for regeneration of corneal surface while CSSCs are used for corneal stromal regeneration. In this review, we have described in detail phenotype and characteristics of LESCs and CSSCs and discussed their therapeutic potential in regenerative ophthalmology.

## 2. Characteristics of LESCs

**Figure 1:**
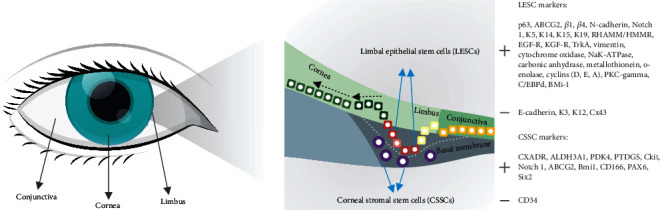
Localization and markers of LESCs and CSSCs.

Corneal epithelia are renewed constantly by the adult stem cells located in the limbal zone making it a unique reservoir or niche of LESCs [9, 10]. Four anatomical sites have been identified as probable LESC locations in humans: palisades of Vogt, limbal epithelial crypts, projections of limbal crypts, and focal stromal projections [11–15]. Small group of LESCs, localized at the basal limbus, retain tritiated thymidine for long periods and are recognized as quiescent cells (Figure 1). Although LESCs are slow cycling cells, they have the high self-renewing and differentiation capacity [16–18]. Since LESCs are derived from neural ectoderm, they may exhibit functional neuronal properties in vitro and may differentiate into neuronal-like cells in vivo, under specific conditions of the microenvironment [19].

At present, there is no currently specific single marker that can be used for identification of LESCs. Combination of stem cell-associated markers, which consisted of a panel of positive and negative markers (Figure 1), can be used to identify putative LESCs [19]. In general, all positive LESC markers are expressed in the basal layers of the epithelium, while their expression in the superficial layers is either reduced or absent. One of the best described positive LESC marker is transcription factor p63, important for epithelial development and differentiation [19, 20]. Holoclone of LESCs expresses high levels of p63; meroclones express low levels of p63, while there is no expression of p63 in paraclones of LESCs. Also, a member of the ATP binding cassette transporter protein, ABCG2, is an additional, well-known marker of LESCs. Integrin *α*9 mediates adhesion to tenascin-C and osteopontin, and it has been localized to small clusters of stem cell-like cells in the limbal basal epithelium [21, 22]. Expression of N-cadherin and Notch 1 on a subpopulation of limbal epithelial basal cells suggests them as possible markers for LESCs [22]. In addition, human LESCs are positive for keratin (K) 5, K14, K15, K19, and vimentin and negative for K3, K12, involucrin, and the gap junction protein Cx43 [22, 23]. RHAMM/HMMR or CD168, an important component of the extracellular matrix, can be used as a negative marker of LESCs as well [24].

The growth factors present in basal cells of limbal epithelium (epidermal growth factor receptor (EGF-R), keratinocyte growth factor receptor (KGF-R), and neurotrophic receptor tyrosine kinase (TrkA)) [21] and proteins associated with cellular metabolic functions which are found in higher concentrations in basal cells of epithelium (Na/K-ATPase, cytochrome oxidase, carbonic anhydrase, alpha-enolase, cyclin D, cyclin E, cyclin A, metallothioneins, and PKC-gamma) may play an important role in LESC metabolism and function [21].

## 3. Characteristics of CSSCs

The presence of self-renewable cells that have the phenotypic characteristics of mesenchymal stem cells (MSCs) and high differentiation potential has been detected in the corneal stroma (Figure 1) and they are called CSSCs [25–31]. Gene array analysis showed that CSSCs have high expression of MSC markers, such as cKIT, Notch 1, ABCG2, BMi1, CD166, PAX6, and Six2 [25]. Moreover, these cells can be expanded 100-fold in a serum-free medium supplemented with ascorbate and insulin when they express keratocyte-specific markers: CXADR, ALDH3A1, PDK4, and PTDGS (Figure 1) [8].

Although both LESCs and CSSCs originate from neural crest-derived MSCs [26], they have different properties and functions in the cornea [27, 28]. LESCs have an important role in regeneration of corneal epithelial surface, while CSSCs are used for regeneration of corneal stroma. The recovered corneal endothelium can be derived from human CSSCs [28], and injection of human CSSCs in lumican-null mice could repair corneal disorders and restore transparency [8], which indicates their therapeutic potential.

## 4. Differentiation of Pluripotent Stem Cells into Corneal Cells

Pluripotent stem cells (PSCs) provide big opportunities for corneal reconstruction by cell-based therapies [32]. Methods for corneal differentiation of pluripotent stem cells are known in the art. Many of these methods are slow or provide only modest differentiation efficiencies. For instance, Japanese researchers in 2012 reported corneal cell differentiation of human induced pluripotent stem cells (iPSCs) on mouse-derived feeder cells taking 12-16 weeks and resulting in a differentiation efficiency of less than 15% based on the expression of CK12 [33], while another group of scientists in 2011 maintained to produce corneal precursor cells by differentiation of mouse iPSCs through cultivation on mouse-derived feeder cells by a method which took a short time [34]. Ahmad et al. [35] used medium conditioned by limbal fibroblasts for culturing human embryonic stem cells (ESCs) previously maintained on a feeder layer of mouse embryonic fibroblasts. This culturing resulted in the loss of pluripotency and differentiation into epithelial-like cells. They reported a differentiation efficiency of 50% on day 5 and 10% on day 21 as measured by expression of proteins CK3/12. Nonetheless, the use of a medium which requires donated limbal cells can be considered problematic. Further, there is a significant biological variation among batches of limbal cells. The differentiation method disclosed is a two-step approach which comprises an induction step, preferably carried out on a suspension culture, at which point the pluripotent stem cells are cultured in the presence of a TGF-beta inhibitor, a Wnt inhibitor, and a fibroblast growth factor, by that producing eye precursor cells [32]. The aforementioned eye precursor cells are then differentiated, in an adherent culture, into corneal epithelial precursor cells in the presence of epidermal growth factor, hydrocortisone, insulin, isoproterenol, and triiodothyronine. Optionally, these corneal epithelial precursor cells may be advanced further into mature corneal epithelial cells or into corneal stratified epithelium [32].

## 5. Therapeutic Potential of LESCs and CSSCs

Thermal or chemical burns, cicatrizing, aniridia, untreated vernal keratoconjunctivitis, and multiple surgeries involving the limbal area can lead to a state of partial or total limbal stem cell deficiency (LSCD) [36].

In patients with unilateral LSCD, autologous limbal transplantation can be utilized to provide surface reconstruction of the cornea [37]. However, this technique requires a large limbal graft from the healthy eye, which can lead to the development of LSCD in that eye [38], and is not applicable to LSCD bilaterally affected patients [39].

**Figure 2:**
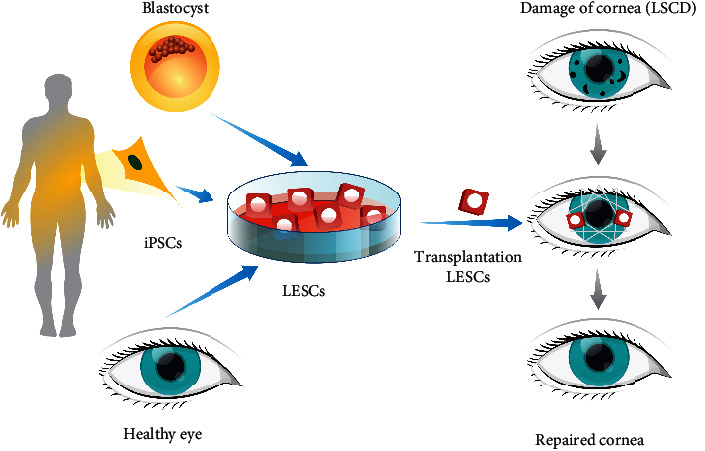
Derivation and therapeutic potential of LESCs.

LESCs can be derived from human ESC or iPSC (Figure 2). Accordingly, autologous tissue-specific cell-based therapy is in focus as a possibly new therapeutic approach for the treatment of LSCD patients. Pellegrini and coworkers were first to report that two patients with unilateral LSCD caused by alkali burns were successfully transplanted with autologous cultivated corneal epithelium, and the results continued for more than two years subsequent to grafting [40]. Following this report, many researchers began investigation of the safety and effectiveness of cultivated limbal epithelial cell transplantation (CLET) [41–43]. As such, one of the clinical efficacies includes the use of the amniotic membrane and fibrin glue utilized as substrates for cultivation of corneal epithelial cells. The amniotic membrane is preferred as it produces cytokines, which allow the survival and self-renewal of limbal stem cells [44]. In addition, Rama and colleagues reported long-term corneal recovery utilizing autologous cultivated LESCs [41]. They demonstrated that permanent repair and a replenishment of the corneal epithelium were accomplished in 76.6% of 107 eyes with LSCD caused by chemical and thermal burns. These results indicated that CLET is a safe and effective procedure. Many factors, such as lack of standardization in terms of patient selection (such as total and partial LSCD used in the same study), cause of LSCD (acquired and congenital), unilateral and bilateral cases of LSCD, source of initial tissue (allo- and autograft transplants in the same study), methods of *ex vivo* expansion (explant or single cell; human amniotic membrane (HAM) or 3T3 fibroblast coculture or both), surgical management (method of superficial keratectomy, the use of a second HAM as a bandage, contact lens protection, or both), and postoperative management (use of HAM or not), represent major obstacles in this field of LESC therapy [44]. Taking previous knowledge and new technologies into consideration, Kolli and coworkers have succeeded in using a nonhuman animal product-free Good Manufacturing Practice- (GMP-) compliant autologous LESC *ex vivo* expansion technique to successfully reverse LSCD within a controlled population and showed 100% success in predefined subjective and objective outcome measures [45]. In addition, they reported, for the first time, the differentiation of hESCs to corneal-like epithelial lineages, providing the first step toward refinement of protocols to produce these cells for potential therapeutic purposes [35, 45].

Based on these data, several clinical trials investigate therapeutic potential of LESCs for the treatment of corneal disorders (Table 1) [46–54]. Results obtained in a phase II study, conducted by Zakaria and coworkers [46], showed that standardized, nonxenogenic culture system, reduced manipulation cultivation, and surgical approach are safe and effective in reducing corneal neovascularization. Tsai and colleagues [49] showed a significant improvement and complete reepithelialization of the corneal surface after two to four days of autologous transplantation of LESCs in all six eyes receiving transplants. In 83% of eyes receiving transplants, mean visual acuity has improved, without recurrent neovascularization and inflammation in the transplanted area during the 15 months of follow-up period. López-García with collaborators investigated histopathologic evolution of the corneal limbus after alkaline burns [50]. In a prospective study of 15 eyes from 12 patients, they demonstrated that the best reepithelialization and stromal regeneration were obtained by autologous limbal transplantation combined with amniotic membrane transplantation. In a clinical study, Holoclar® is the only licensed autologous LSC product in Europe for the treatment of patients with unilateral and bilateral (one eye partial) LSCD caused by ocular surface buns [51].

CSSCs, as newly identified corneal stem cells, provide hope and opportunity for the treatment of so far incurable condition of the cornea. Although preclinical studies suggest therapeutic potential of CSSCs [8, 27, 28], there are currently no clinical trials that use these cells. Further studies are necessary to develop optimized protocols for their isolation and characterization as well as reliable assays to evaluate their therapeutic potential.

## 6. New Paradigm: Cell-Free Stem Cell Therapy

The effects of MSCs are related to soluble secreted factors that are involved in the process of tissue wound repair, inflammation, angiogenesis, and immune response [55]. Most MSCs have the affinity to accumulate within the filtering organs, i.e., lungs, liver, and spleen, after intravenous delivery. However, MSCs can regulate tissue repair, after achieving only minimal engraftment at the site of tissue injury [56]. Subconjunctival MSC injection to alkali-injured corneas promoted corneal wound healing, despite the MSCs remaining in the subconjunctival space [57]. Additionally, topical administration of MSCs or conditioned MSC media to a murine corneal epithelial wounding model has shown benefits in terms of attenuating corneal inflammation, reducing neovascularization, and promoting wound healing [58]. Taking into account the previous results, it can be concluded that MSC exert their effect through a paracrine mechanism, rather than direct cell replacement, since most of the MSCs were retained in the corneal stroma rather than the epithelium. These effects are most likely mediated through secreted soluble factors released from MSCs in the form of extracellular vesicles or exosomes [59, 60].

Exosomes are produced by cultured cells and subsequently released into the conditioned media. Different methods of exosomes isolation have been established, including differential centrifugation, density gradient centrifugation, filtration, size exclusion chromatography, polymer-based precipitation, immunological separation, and sieving [61]. The size of exosomes is restricted by multivesicular bodies in the parental cells and ranges from 30 nm up to several hundred nm in diameter. The luminal content of exosomes contains proteins, lipids, and nucleic acids (DNA, mRNA, miRNAs, and long noncoding RNAs), although the exact composition and content of the exosomal cargo released by different cell types are difficult to determine, due to differences within cellular environments [62].

MSC-derived exosomes (MSC-Exo) can encapsulate and transfer biomolecules that have effects on cell and tissue metabolism, including differentiation, inflammation, angiogenesis, immunosuppression, neurogenesis, and synaptogenesis [63, 64]. The periocular injection of human umbilical cord MSC-Exo into an experimental rat autoimmune uveitis (EAU) model decreases inflammation by downregulating MCP1/CCL21- and MYD88-dependent pathways [65]. The cells expressing Gr-1, CD68, CD161, CD4, IFN*γ*, and IL17, respectively, served to restore retinal function. Intravitreal injection of exosomes from umbilical or adipose MSC cultures modifies the inflammation and improves visual function in retinal injury induced by laser, through the inhibition of MCP1, ICAM-1 (intercellular adhesion molecule-1), and TNF*α* [66]. Hyperglycemia-induced retinal inflammation in diabetic rats was also shown to be improved by an intravitreal injection of human umbilical cord MSC-Exo, as well as an intravitreal injection of umbilical cord MSC-Exo in blue light-induced retinal damage [67]. The latter showed a dose-dependent suppression of choroidal neovascularization through downregulation of VEGFA and inhibition of the NF*κ*B pathway, possibly by miR-16 transfer [68]. Ganglion cell growth can be stimulated by intravitreal injection of bone marrow MSC-Exo cells in a rat optic nerve crush model, through argonaute-2 signaling, which stabilized miR-16 activity from RNase digestion [69]. Given the fact that intravenous MSC administration caused similar recovery of retinal functions in EAU and laser-induced retinal injury models, it can be concluded that the therapeutic effects of MSC may be mediated through MSC-Exo action [70]. A recent clinical trial has shown that patients with refractory macular holes had anatomical and functional recovery after intravitreal injection of human umbilical cord MSC-Exo. Nevertheless, one patient experienced an inflammatory reaction [71].

This cell-free strategy may also have a significant impact on corneal wound repair, through stimulation of different factors that modulate inflammation, angiogenesis, and tissue regeneration. Few studies have demonstrated the therapeutic functions of soluble factors from MSC-Exo on corneal wound models. Cultivation of rabbit corneal stromal cells, and rabbit adipose MSC-Exo, has led to greater proliferation, along with the deposition of new ECM proteins (including collagens). Topical CSSC-derived exosomes can suppress corneal inflammation and corneal scarring through the inhibition of neutrophil infiltration. Moreover, murine corneal epithelial wound healing can be promoted by exosomes from human corneal mesenchymal stromal cells [72]. Umbilical cord MSC-Exo carrying *β*-glucuronidase reduced the accumulated glycosaminoglycans in a mouse mucopolysaccharidosis model, thereby reducing corneal haze. These data have highlighted the potential for the therapeutic use of MSC-Exo in ocular surface diseases and congenital corneal metabolic disorders [73].

## 7. Opportunities and Challenges in Regenerative Ophthalmology

From the earliest concepts such as replacement of the opaque cornea to corneal wound healing and regeneration, ophthalmologists and material scientists across the world have faced a collection of challenges [74, 75]. Advances in visualization techniques and histology have made significant progress in the fundamental understanding of cornea structure and its microenvironment. As a result of this valuable information and nanotechnology advances, therapeutic strategies in devastating corneal diseases have turned from corneal replacement into corneal wound healing and regeneration [76]. Ergo, studies on the limbus zone and immune and angiogenic privilege have attracted more attention. In addition, the exploration of cell signaling in the natural process of wound healing and the attempts to mimic this process have opened new horizons in corneal disease treatment.

A large number of the suggested treatments have shown promising results for wound healing at the ocular surface, and entire thickness dystrophies were neglected. At the same time, in order to reduce transplantation of a donor cornea, tissue engineering of the whole thickness of the cornea must be considered. Corneal stromal and endothelium tissue engineering has recently shown noticeable progress [77]. Nonetheless, more focus should be on biomimetic strategies, such as employing a combination of cell signaling agents with tissue engineering. Rho-kinase (ROCK) inhibitor is a serine/threonine protein kinase that participates in regulating cell signaling route. In recent past, ROCK has been announced as an innovative therapeutic agent for corneal endothelial dystrophy [78]. The combination of these approaches can be a promising method for visual rehabilitation in patients suffering from corneal dystrophies.

So far, most studies have worked on presenting new materials and biochemical approaches in corneal wound healing and regeneration, while putting accent on physical properties of these approaches could be a leap in this area. For instance, Long et al. have tried to use a cross-linking agent in collagen membrane to regulate collagen fibril spacing and hence improve optical clarity of collagen and increase permeability of neurites [79]. Accordingly, advances in visualization techniques will help in the improvement of corneal physical structure identification that, in combination with material science, will lead to new perceptions in the typical treatment approaches. Slit-lamp biomicroscopy, optical coherence tomography (OCT), in vivo confocal fluorescence microscopy, and full-field optical microscopy are part of visualization techniques which help to quantify corneal architecture [80, 81]. As stated in previous studies, investigation on visualization methods would expand corneal medical treatments.

Considering the exceptional role of stem cells in tissue regeneration, a large part of future studies is expected to focus on the deployment of stem cells on corneal wound healing and regeneration [82]. A certain number of studies have been done to isolate and characterize multipotent stem cells from different tissues in order to use their great potential in regenerative medicine. Bone marrow-derived mesenchymal stem cells [83], human umbilical cord mesenchymal stem cells [84], postnatal periodontal ligament [85], and limbal stem cells [86] are recently studied stem cells sources in corneal wound healing and regeneration. Saghizadeh et al. [30] have recently reviewed all major stem cell usage in corneal wound healing. Contrarily, developing innovative methods to produce 3D tissue-like architecture has allowed mimicking the microarchitecture and physiology of the native cornea. In this regard, 3D microfabrication methods are promising approaches in designing cornea substitutes [87, 88]. Amidst additive manufacturing methods, study on bioprinting and the development of bionics provides a great promise in relation to the fabrication of human corneal substitutes that mimic the structure of native corneal tissues [89, 90].

## 8. Conclusion

The concept of corneal stem cells has greatly enhanced the understanding of corneal epithelial proliferation, migration, and recovery. This has also contributed directly to improve medical and surgical management of a wide range of ocular surface disorders. On the other hand, control of scar tissue formation is of great importance for corneal regeneration and recovery of eyesight. However, it should be noted that there are still several problems including insufficient data regarding safe and successful LESC and CSSC engraftment in the human cornea and their long-term efficacy, which limit their capacity to be used as a main treatment approach for corneal regeneration.

## Figures and Tables

**Table 1:** 

	Study title	Conditions	Interventions	Status	Locations
1	The application of cultured cornea stem cells in patients suffering from corneal stem cell insufficiency	Unilateral limbal stem cell insufficiency	Procedure: transplant of cultured limbal stem cells on the cornea	Terminated	National Taiwan University Hospital, Department of Ophthalmology, Taipei, Taiwan

2	The application of oral mucosal epithelial cell sheets cultivated on amino membrane in patients suffering from corneal stem cell insufficiency or symblepharon	Limbal insufficiency Symblepharon	Procedure: cultured oral mucosa cell sheet transplantation	Terminated	National Taiwan University Hospital, Department of Ophthalmology, Taipei, Taiwan

3	Limbus-derived stem cells for prevention of postoperative corneal haze	Corneal scars and opacities	Biological: stem cells Other: vehicle	Recruiting	LV Prasad Eye Institute, Hyderabad, Telangana, India

4	The improvement of limbal stem cell deficiency (LSCD) in unilateral stem cell damage by amniotic membrane extract eye drop (AMEED)	Limbal stem cell deficiency (LSCD)	Biological: amniotic membrane extract eye drop (AMEED)	Completed	—

5	Efficacy and safety of autologous cultivated limbal stem cells transplantation (ACLSCT) for restoration of corneal epithelium in patients with limbal stem cell deficiency	Limbal stem cells deficiency	Biopsy from donor eye Implant of Holoclar Ophtalmologic examination (and 5 more)	Recruiting	Hospital San Raffaele, Milan, Italy

6	Stem cells therapy for corneal blindness	Corneal injuries Corneal burns Corneal scars and opacities	Biological: ex vivo cultivated limbal stem cell pool	Unknown	LV Prasad Eye Institute, Hyderabad, Telangana, India

7	Autologous cultured corneal epithelium (CECA) for the treatment of limbal stem cell deficiency	Limbal stem cell deficiency	Procedure: surgical transplantation of CECA	Recruiting	CIUSSS de l'Est de l'île de Montréal, Quebec, Canada Centre Universitaire d'Ophtalmologie CHU de Québec-HSS Québec, Canada

8	Autologous adipose-derived adult stem cell transplantation for corneal diseases	Hereditary corneal dystrophy Keratoconus	Procedure: lipoaspiration Procedure: transplantation	Unknown	Optica General, Saida, Lebanon

9	Corneal epithelial autograft for LSCD	Limbal stem cell deficiency	Corneal epithelial and limbal conjunctival autograft Femtosecond laser and diamond knife	Recruiting	Zhongshan Ophthalmic Center, Sun Yat-sen University, Guangzhou, Guangdong, China

10	Umbilical cord mesenchymal stem cells injection for ocular corneal burn	Ocular corneal burn	Biological: human umbilical cord mesenchymal stem cells Biological: placebo	Unknown	The First Affiliated Hospital of Jinan University, Guangzhou, Guangdong, China

11	Follow-up study after ACLSCT for restoration of corneal epithelium in patients with LSCD due to ocular burns	Limbal stem cell deficiency due to ocular burn	Procedure: ophthalmologic examinations Other: digital pictures Other: QoL questionnaires	Recruiting	Hospital San Raffaele, Milan, Italy

12	Corneal epithelium repair and therapy using autologous limbal stem cell transplantation	Corneal disease Pterygium Myopia Hyperopia	LSCs and amniotic membrane (modified technique); amniotic membrane only (traditional technique); PRK, LSCs, and amniotic membrane (modified technique) (and 4 more)	Unknown	Zhongshan Ophthalmic Center, Sun Yat-sen University, Guangzhou, China

13	Corneal epithelial stem cells and dry eye disease	Dry eye syndromes Dry eye ocular inflammation (and 3 more)	Other: corneal epithelial stem cell transplant	Enrolling by invitation	Rush Eye Associates, Amarillo, Texas, United States

14	Efficacy of cultivated corneal epithelial stem cell for ocular surface reconstruction	Severe ocular surface damage Limbal deficiency	Procedure: cultivated limbal transplantation	Completed	Pinnita Prabhasawat, MD, Bangkok, Thailand

15	Corneal epithelial allograft from living-related donor for LSCD	Limbal stem cell deficiency	Procedure: corneal epithelial allograft Procedure: limbal conjunctival allograft Device: femtosecond laser Device: diamond knife	Completed	Zhongshan Ophthalmic Center, Sun Yat-sen University, Guangzhou, Guangdong, China

16	The treatment of human bone marrow mesenchymal stem cells in ocular corneal burn	Chemical burns	Other: human bone marrow MSC	Completed	—

17	Clinical trial on the effect of autologous oral mucosal epithelial sheet transplantation	Limbal stem cell deficiency Stevens-Johnson syndrome Ocular cicatricial pemphigoid Chemical burn	Biological: cultivated oral mucosal epithelial sheet transplantation	Available	Seoul National University Hospital, Seoul, Republic of Korea

18	Cultivated limbal epithelial transplantation (CLET) for limbal stem cell deficiency (LSCD)	Limbal stem cell deficiency	—	Not yet recruiting	IOBA, Valladolid, Spain

19	Limbal stem cell deficiency (LSCD) treatment with cultivated stem cell (CALEC) graft	Limbal stem cell deficiency	Procedure: biopsy to collect limbal epithelial stem cells that will be cultivated into a graft Biological: cultivation of limbal epithelial cells into a graft Procedure: CALEC transplant Procedure: CLAU	Recruiting	Massachusetts Eye and Ear Infirmary, Boston, Massachusetts, United States
